# Pathway focused protein profiling indicates differential function for IL-1B, -18 and VEGF during initiation and resolution of lung inflammation evoked by carbon nanoparticle exposure in mice

**DOI:** 10.1186/1743-8977-6-31

**Published:** 2009-12-02

**Authors:** Koustav Ganguly, Swapna Upadhyay, Martin Irmler, Shinji Takenaka, Katrin Pukelsheim, Johannes Beckers, Eckard Hamelmann, Holger Schulz, Tobias Stoeger

**Affiliations:** 1 Comprehensive Pneumology Center, Institute of Lung Biology and Disease, Helmholtz Zentrum München, German Research Center for Environmental Health, Ingolstaedter Landstrasse 1, Neuherberg/Munich, D85764, Germany; 2 Institute of Experimental Genetics, Helmholtz Zentrum München, German Research Center for Environmental Health, Ingolstaedter Landstrasse 1, Neuherberg/Munich, D85764, Germany; 3 Center of Life and Food Sciences, Technical University Munich, Weihenstephan, Alte Akademie 8, Freising, 85354, Germany; 4 Department of Pediatric Pneumology and Immunology, Charité Universitätsmedizin Berlin, Augustenburger Platz 1, D-13353 Berlin, Germany; 5 Department of Pediatrics, Ruhr-University Bochum, Alexandrinenstraße 5, 44791 Bochum, Germany

## Abstract

**Background:**

Carbonaceous nanoparticles possess an emerging source of human exposure due to the massive release of combustion products and the ongoing revolution in nanotechnology. Pulmonary inflammation caused by deposited nanoparticles is central for their adverse health effects. Epidemiological studies suggest that individuals with favourable lung physiology are at lower risk for particulate matter associated respiratory diseases probably due to efficient control of inflammation and repair process. Therefore we selected a mouse strain C3H/HeJ (C3) with robust lung physiology and exposed it to moderately toxic carbon nanoparticles (CNP) to study the elicited pulmonary inflammation and its resolution.

**Methods:**

5 μg, 20 μg and 50 μg CNP were intratracheally (i.t.) instilled in C3 mice to identify the optimal dose for subsequent time course studies. Pulmonary inflammation was assessed using histology, bronchoalveolar lavage (BAL) analysis and by a panel of 62 protein markers.

**Results:**

1 day after instillation of CNP, C3 mice exhibited a typical dose response, with the lowest dose (5 μg) representing the 'no effect level' as reflected by polymorphonuclear leucocyte (PMN), and BAL/lung concentrations of pro-inflammatory proteins. Histological analysis and BAL-protein concentration did not reveal any evidence of tissue injury in 20 μg CNP instilled animals. Accordingly time course assessment of the inflammatory response was performed after 3 and 7 days with this dose (20 μg). Compared to day 1, BAL PMN counts were significantly decreased at day 3 and completely returned to normal by day 7. We have identified protein markers related to the acute response and also to the time dependent response in lung and BAL. After complete resolution of PMN influx on day 7, we detected elevated concentrations of 20 markers that included IL1B, IL18, FGF2, EDN1, and VEGF in lung and/or BAL. Biological pathway analysis revealed these factors to be involved in a closely regulated molecular cascade with IL1B/IL18 as upstream and FGF2/EDN1/VEGF as downstream molecules.

**Conclusion:**

Considering the role of VEGF, FGF2 and EDN1 in lung development and morphogenesis together with the lack of any evident tissue damage we suggest a protective/homeostatic machinery to be associated in lungs of stable organisms to counter the CNP challenge as a precautionary measure.

## Introduction

Various epidemiological and clinical studies have shown the correlation between ambient air particle concentration and adverse respiratory health effects throughout the industrialized world [1-3]. It is considered that individuals with impaired lung physiology are at higher risk to various respiratory diseases like chronic obstructive pulmonary disease (COPD) which are accelerated due to chronic exposure to environmental stressors like ultrafine particles/nano particles [4-6]. Therefore, individuals having a favorable lung physiology are considered to be at lower risk to various obstructive lung diseases probably due to efficient control of inflammatory and/repair processes. In addition to the widely accepted adverse health effects of anthropogenic airborne particulate pollution, the increasing use of engineered nanopaticles in all spheres of life has also become a new source of human exposure [7]. Carbon nanoparticles (CNP) regardless of their different sources, as combustion-derived nanoparticles or carbon based engineered nanoparticles, are of toxicological interest given their nanosized dimensions, with properties not displayed by their macroscopic counterparts. The pulmonary deposition efficiency of inhaled sub-100 nm particles, along with their large surface areas, is considered to be important in driving the emerging health effects linked to respiratory toxicity [8,9].

To approach these issues experimentally we applied a standardized exposure scenario to C3H/HeJ (C3) mice with favorable lung physiology among several inbred mouse strains and defined it as our physiological base [10,11]. Moderately toxic and physically-chemically well characterized carbon nanoparticles (CNP) [12] were used as the exposure material and defined it as the toxicological base. Carbon black is an ingredient in rubber, plastics, inks and paints with an annual production of 10 million tones [13] indicating its wide usage and potentially massive exposure in day to day life among people of various working class. CNP also constitutes the core of combustion derived particles [14] and represents relevant surrogates for exhaust particles from modern diesel engines [15,16]. Since particle surface area and chemical composition have been described to drive particle related toxicity [12,17,18] it is important to perform experiments with well characterized particles like the here used CNP so as to minimize the effects of uncharacterized chemical composition. CNP constitutes of 98% pure carbon [19] and therefore exhibit no evidence for any bioactivation of organic compounds. Hence the inflammatory efficacy of CNP exclusively depends on their innate surface reactivity [15].

A single exposure to ultrafine carbon black particles (here called CNP) has been shown to cause a dose dependent but transient inflammatory and cytotoxic response in the lungs of rodents [20,21]. The goal of the present study is to directly focus on this transient nature of the inflammatory response by using the two defined bases (physiological and toxicological) and analyze the time course response following CNP exposure in lung tissue using a comprehensive panel of well characterized protein markers. At this point our first aim was to characterize and determinate the acute dose response to assess the validity the applied protein markers and to identify the most suitable CNP dose to be used for the subsequent time course analysis. Since previous experiments indicated that the inflammatory response is to the greatest extent resolved at day 7 after exposure, we limited our analysis to day 3 and 7. Response to foreign material deposited on the cellular surface of the lungs due to particle inhalation is multidimensional and includes the 'innate immune response', the 'adaptive response' and the 'tissue repair and remodeling mechanism'. The central feature of the innate immune response is the recruitment and activation of granulocytes also called polymorphonuclear leucocytes (PMN) at the site of stimulus [22], carbon nanoparticles (CNP) in this case. The innate response is typically triggered when pathogens like microbes are identified by pattern recognition receptors, or when damaged, injured or stressed cells send out alarm signals. Innate response involves neutrophil, granulocytes, macrophages, platelets and the complement system, but also eosinophils, mast cells, basophils, and natural killer cells [23]. The adaptive/specific immunity uses antigen specific receptors on the T and B cells to drive targeted effector responses. The adaptive immunity involves the two way process of differentiation and activation with the main modulators being lymphocytes, alveolar macrophages and epithelial/endothelial cells [23]. The tissue response/repair and remodeling mechanism involves the interaction of various growth factors and fibroblasts to restore homeostasis [24].

To investigate the complex response due to CNP exposure standard procedures of cell differential counting and protein estimation from bronchoalveolar lavage (BAL) combined with lung histology has been used to assess the degree of inflammation. To gain insight into the underlying molecular mechanism we applied a wide panel of 62 protein markers covering a broad spectrum of biological processes (Additional file 1; Table S1), considered to be important in relation to particle exposure. Our panel of markers are known to play important roles in the following key processes of lung tissue: **i) **Initiation and amplification of inflammation **ii) **Induction of T-cell independent macrophage activation **iii) **Regulation of dendritic cell maturation and differentiation, and **iv) **Regulation of T-cell activation and differentiation as described by [24]. The highlight of our work has been the identification of protein markers for acute-dose and time-course response following CNP exposure. We were able to associate a closely orchestrated pathway with IL1B/IL18 as the upstream and FGF2/EDN1/VEGF as the downstream molecules thatis active even after the complete resolution of PMN influx. Considering the role of VEGF, FGF2 and EDN1 in lung development and morphogenesis together with the lack of any evident tissue damage we suggest a protective/homeostatic machinery to be activated in lungs of stable organisms to counter the CNP challenge as a precautionary measure.

## Results

The experimental animal groups were designed so as to obtain an acute dose-response relationship [5 μg/day 1, 20 μg/day 1 and 50 μg/day 1] after CNP instillation and a subsequent time course response for the moderate dose of 20 μg [20 μg/day 1, 20 μg/day 3, 20 μg/day 7] in C3 mice following intra tracheal (i.t) instillation of CNP. Because no significant differences where observed between untreated controls and sham (vehicle) exposed controls all CNP exposed groups were compared to sham exposed controls.

### BAL cell differentials and protein leakage

**Figure 1:**
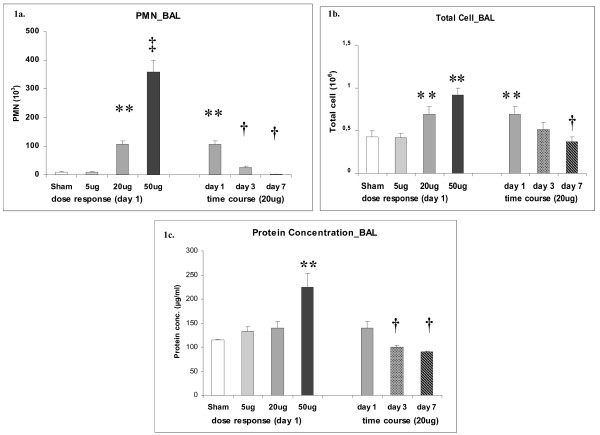
**Bronchoalveolar lavage fluid (BAL) cell differentials and protein concentration**. a) Dose dependent influx and time dependent resolution of polymorphonuclear leukocytes (PMN) in the BAL following intratracheal (i.t.) instillation of carbon nanoparticles (CNP) particles in the C3H/HeJ (C3) mice. (n = 7 animals/experimental group). b) Total cell count in the BAL of C3 mice following i.t. instillation of CNP particles. c) Total protein concentration in the BAL of C3 mice showing a dose dependent increase and time dependent decrease following i.t instillation of CNP. (**) Significantly different with respect to (w.r.t) both sham control and 5 μg exposed; (†) significantly different w.r.t 20 μg exposed at day 1; (††) significantly different w.r.t 20 μg at day 1, 5 μg and scham control.

A dose dependent increase of PMN was detected on day 1 after CNP instillation in BAL samples of C3 mice. No significant induction of PMN has been observed following i.t. instillation of 5 μg CNP (9.41 ± 3.16 × 10E3 PMNs) compared to the control (9.23 ± 3.05 × 10E3 PMNs). A significant influx of PMN has been detected following 20 μg (105.53 ± 12.37 × 10E3 PMNs) and 50 μg (358.24 ± 41.91 × 10E3 PMNs) CNP instillation in a dose dependent manner (Figure: 1a). BAL samples of 20 μg CNP instilled lungs revealed significantly reduced PMN counts after 3 days (25.78 ± 4.96 × 10E3 PMNs) and 7 days (2.54 ± 0.57 × 10E3 PMNs) compared to that of day 1. At day 7 the PMN count reached to the baseline values indicating complete resolution of the neutrophil influx related inflammation following 20 μg of CNP instillation. A similar trend of events was also observed in the total BAL cell count (Figure: 1b), since the overall number of other cell types like macrophages and lymphocytes where not significantly changed upon dose or time response (data not shown).

Compared to sham exposed mice, only the instillation of the highest dose (50 μg/mouse) caused a significant increase in total BAL protein concentration (224.1 ± 29.0 μg/ml versus 114. 4 ± 2.2 μg/ml) 1 day after CNP instillation, indicating capillary leakage at this concentration i.e. alveolar barrier injury at this dose (Figure 1c). Time course investigation of 20 μg instilled lungs however revealed a significant decrease of BAL protein concentrations from day 1 (140.0 ± 13.2 μg/ml) to day 3 (99.5 ± 4.8 μg/ml) and day 7 (89.8 ± 2.2 μg/ml).

### Histopathology

Histopathological analysis of the paraffin embedded lung sections (n = 4) showed a typical dose dependent accumulation of particle laden macrophages and also a time dependent clearance as reported in many studies. The degree of accumulation of particle laden macrophages can be categorized as follows: 5 μg/day 1 (very slight/slight); 20 μg/day 1 (slight/moderate); 50 μg/day 1 (moderate; focal but marked); 20 μg/day 3 (slight); 20 μg/day 7 (very slight/slight). In 50 μg/day 1 samples inflammatory cell infiltration (PMN) was clearly visible whereas at 20 μg/day 1 slight PMN infiltration was visible.

### Molecular analysis for lung and BAL compartment

Marker selection: To select useful molecular markers following CNP i.t instillation from our panel we have defined a set of criteria based on which they were selected for further analysis/discussion.

• Markers showing a clear dose response following exposure to CNP (5 μg, 20 μg and 50 μg) in either lung homogenate and/BAL were only considered. We defined dose response as a significant increase/decrease of the expression level of a particular marker with the ascending CNP dosage compared to the sham control. Undetectable markers were not considered.

• For BAL, markers detected at/above sensitivity level (see methods) in the pooled samples were only considered. Markers below sensitivity level were not considered due to lack of scope of reproducibility in multiple independent samples (n = 4 BAL samples were pooled/experimental group).

• For lung homogenate markers below sensitivity levels were considered as the results were reproducible in multiple independent samples and were measureable in all cases (n = 4).

Additional file 1; Table S1 depicts the association of all the 62 markers measured to the explained criteria along with their respective gene symbol, Entrez identification number, associated gene ontology terms [GO; Biological process; according to mouse genome informatics (MGI) database] and the corresponding sensitivity level for measurement. Applications of these criteria lead us to exclude 11 markers from the overall analysis from our panel of 62 markers from both lung and BAL. The exclusion list from both lung and BAL analysis consists of the following: APOA1, CD40, Haptoglobin, IgA, IL-17, SGOT, HO-1, Osteopontin and Myoglobin for not exhibiting any response; IL-3 and GST-α being not detectable. Therefore 51 out of 62 measured proteins have been further analyzed in the *lung homogenate *of which 36 were above and 15 were below the sensitivity level. We have analyzed multiple (n = 4) independent lung homogenate samples which shows a strong homogeneity of the data within each experimental group and therefore included the markers below sensitivity level also in our analysis. In the *pooled BAL *samples 21 out of 62 markers were detected at or above the sensitivity level. All of these 21 BAL markers exhibited a clear dose response after day 1 with the highest levels being detected at 50 μg dose in all cases. All values were compared to sham control samples.

### Lung compartment

**Figure 2:**
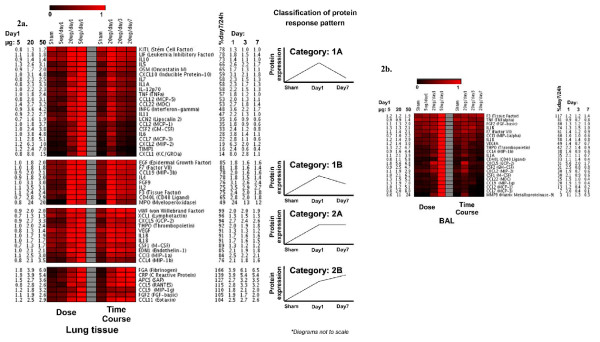
**Heatmap representation of protein concentration levels in lung homogenate and bronchoalveolar lavage (BAL) following intratracheal instillation of carbon nanoparticles (CNP) in C3H/HeJ (C3) mice**. Data shown are normalized (highest value is set to "1") for **(a) **dose and time response for the lung; and **(b) **dose and time response for the BAL, respectively. Red color indicates increasing concentration levels and official protein symbols and the fold inductions for each protein at different dose and time are shown. A schematic graphical representation of the markers categorized as category 1A, 1B, 2A, and 2B in the lung as calculated by the ratio [percent day 7/day 1] is shown and are explained in the results section.

Response of the protein markers from the lung homogenate are presented as heat map (Figure 2a). We could detect four distinct categories of response pattern over time as explained in the following section.

#### Category 1A

This category consists of 22 markers (Figure 2a). The list includes: KITL, LIF, IL10, IL5, OSM, CXCL10, IL7, IL1A, IL12p70, TNFα, CCL12, CCL22, IFNγ, IL11, LCN2, CCL2, CSF2, IL6, CCL7, CXCL2, TIMP1, and CXCL1 (arranged serially as in figure 2A). Concentration levels of these markers at day 7 were significantly decreased compared to the increased levels detected at 20 μg/day 1 and the levels reached to basal levels detected in controls. At 20 μg/day 1 concentration levels were significantly elevated compared to controls and/5 μg/day 1. MMP9 and VCAM1 exhibited a significantly increased response only at 50 μg/day 1 and were therefore not considered for the time course analysis and not represented in the heatmap.

#### Category 1B

This category consists of 9 markers (Figure 2A). The list includes: EGF, F7, CCL19, IL4, FGF9, IL2, F3, CD40L, and MPO (arranged serially as in figure 2a). Concentration levels of these markers at day 7 are significantly decreased compared to the increased levels detected at 20 μg/day 1 but the levels are still higher than the basal levels detected in control. At 20 μg/day 1 concentration levels were significantly increased compared to control and/5 μg/day 1.

#### Category 2A

This category consists of 11 markers (Figure 2a). The list includes: vWF XCL1, CXCL5, THPO, VEGF, IL1B, IL18, CSF1, EDN1, CCL3, and CCL4 (arranged serially as in figure 2A). Concentration levels of these markers at day 7 remains equivalent to the increased levels detected at 20 μg/day 1. i.e. concentration levels were not significantly different compared to 20 μg/day 1 and 20 μg/day 3. At 20 μg/day 1 concentration levels were significantly higher compared to control and/5 μg/24 h.

#### Category 2B

This category consists of 7 markers (Figure 2a). The list comprises of: fibrinogen, CRP, APCS, CCL5, CCL9, and FGF2 (arranged serially as in figure 2a). These markers exhibit a consistent trend of ascending response at day 7 as calculated by the ratio [percent day 7/day 1] but the concentrations levels were not significantly different compared to 20 μg/day 1 and/20 μg/day 3. At 20 μg/day 1 concentration levels were significantly increased compared to sham and/5 μg/day 1.

### BAL compartment

F3, TNFα, FGF2, IL1B, F7, CCL3, IL18, VEGF, THPO, CCL4, IL1A, CD40L, CXCL5, CSF2, CXCL2, CSF1, CCL22, CCL9, CCL2, , CCL7, MMP9 were detected at/above the sensitivity level and exhibited a clear dose response in the pooled BAL samples as represented in the heatmap (Figure 2b). Most of the BAL markers except F3, CD40L, IL1B, IL18, CCL9 and FGF2 exhibited the expected descending response pattern with time (i.e *similar to category 1A in lung*). CXCL5 exhibited a clear descending response with time but it was still 1.7 folds higher on day 7 compared to the sham control (i.e *similar to category 1B in lung*). CXCL5 was also observed to the most sensitive BAL marker being the only to be differentially regulated at 5 μg/day 1. It was upregulated by 1.8 fold at this dose. Highest levels of F3 concentration was detected on day 7 after 20 μg CNP instillation when PMN influx was resolved (i.e *similar to category 2B in lung*). Interestingly CD40L and IL1B concentration levels in the BAL were detected at highest levels on day 3 after 20 μg CNP instillation in contrast to the other markers which peaked at day 1 (i.e. *delayed response*). Concentration levels of CCL9, FGF2 and IL-18 remained equivalently high to day 1 levels also on day 3 following CNP instillation and were observed to be reduced by day 7 (i.e. *extended elevated levels but limited to day 3*). It was interesting to note that several markers were detected at down regulated (0.6 fold or less) levels on day 7 compared to the sham control. They include: CCL3, CCL4, CD40L, CXCL2, CSF1, CCL22, CCL9, CCL2, CCL7, and MMP9. In fact CCL2 and CCL7 were already detected at markedly down regulated levels already on day 3. MMP9 was detected at 0.6 fold lower compared to sham control at 5 μg/day 1.

These findings in the lung and BAL compartment indicate the existence of a complex interacting and regulatory mechanisms in between these markers which may be different between the lung and BAL compartment and may also be due to the pleotrophic functional nature of these proteins.

### Time course markers

On this basis when we curated the molecular data in the lung homogenate and BAL we were able to identify a set of markers that are sensitive for the acute response at the day 1 time point, and another set of markers which are essential even after complete resolution of neutrophil influx related inflammation (day 7). The later molecular events can be regarded as plausible protective machinery in place up to 7 days following exposure to moderately toxic CNP in a mouse strain with robust lung physiology as we did not detect any obvious tissue injury/damage. These time course markers may be considered to have pathophysiological relevance as they exhibit elevated levels also on day 7 plausibly for maintaining homeostasis.

**Figure 3:**
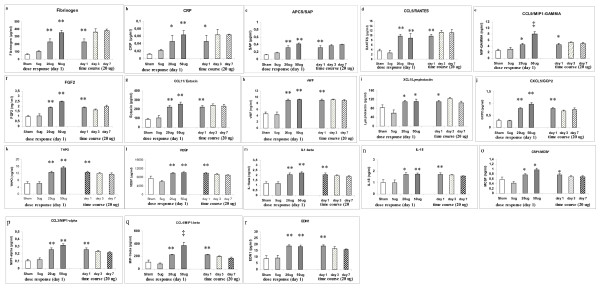
**Time course response markers (categories 2A and 2B) in the lung homogenate following intratracheal (i.t.) instillation of carbon nanoparticles (CNP) in C3H/HeJ (C3) mice**. (n = 4 animals/experimental group). **(a-r) **lists the markers which showed a typical dose response but remained at elevated levels (not significantly different compared to 20 μg/day 1) even after day 7 following a significant induction at day 1 due to 20 μg i.t. instillation of CNP. At day 7 neutrophil influx related inflammation was completely resolved. Gene symbols are used as shown in additional file 1; Table S1. (**) Significantly different with respect to (w.r.t) both scham (Sham) control and 5 μg exposed; (*): Significantly different w.r.t scham control or 5 μg exposed; (†) significantly different w.r.t 20 μg exposed at day 1; (††) significantly different w.r.t 20 μg at day 1, 5 μg and sham control.

18 markers represented in categories 2A and 2B in the lung compartment have been identified to be important in this context for being detected at elevated levels on day 7. They are as follows: fibrinogen, CRP, APCS, CCL5, CCL9, FGF2, CCL11, vWF, XCL1, CXCL5, THPO, VEGF, IL1B, IL18, CSF1, EDN1, CCL3, and CCL4. Individual response pattern of these markers are represented in figure (3a-r). We have also identified 6 BAL markers (F3, CD40L, FGF2, IL1B, IL18 and CCL9) to be important for time course following 20 μg CNP instillation as they exhibited a delayed response or extended elevated levels. Although CD40L and F3 exhibited a significant descending trend at day 7 in the lung compartment, their levels were quite high compared to baseline levels. Therefore the expression trend of these 6 markers in the lung compartment essentially holds true also in the BAL compartment. However CSF1, CCL3, CCL4 and VEGF differ in their time course regulation pattern between BAL compartment and lung compartment following 20 μg CNP instillation. These 4 markers remains significantly elevated at day 7 in the lung whereas they are reduced in the BAL at this time point.

### Biological pathway Analysis

**Figure 4:**
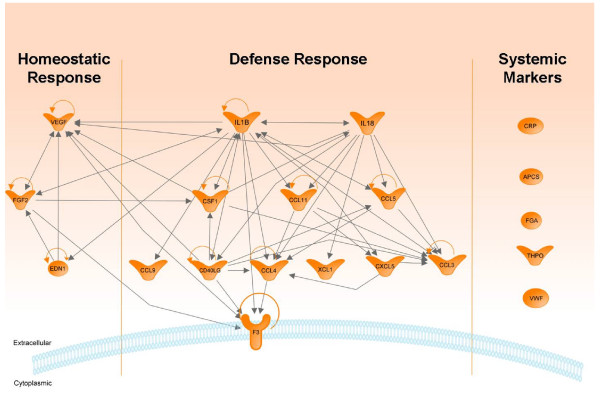
**Pathway analysis of proteins upregulated during time course analysis in lung or bronchoalveolar lavage fluid (BAL) following intra tracheal instillation of 20 μg carbon nanopaticles (CNP)**. At day 7, when complete resolution of neutrophilic influx has taken place all of these markers were detected at significantly elevated concentration compared to their base line levels following their initial upregulation during the acute response phase (day 1). The predicted molecular network suggest a central role of IL1B (IL: Interleukin) and IL18 in the process. Regulation of vascular endothelial growth factor (VEGF) through the endothelin1 (EDN1) and basic fibroblast growth factor (FGF2) network as the end product in the cascade to restore homeostasis. Elevated levels of several systemic factors suggest their role locally for maintaining/restoring homeostasis. Gene symbols are used as shown in Additional file 1; Table S1.

Based on the identification of markers which remained in elevated concentrations for an extended time period in lung (at day 7) and BAL (at day 3/day 7) we performed an extensive literature study which revealed that IL1B and IL18 act as the central players; and regulation of VEGF via EDN1 and FGF2 as the ultimate events. This observation is also illustrated in the pathway analysis (Figure 4) for the time course response markers from lung and BAL. The pathway analysis involved the following markers: CCL5, CCL9, FGF2, CCL11, XCL1, CXCL5, VEGF, IL1B, IL18, CSF1, EDN1, CCL3, CCL4, F3 and CD40L for homeostatic and defence responses. We preferred to group the classical systemic markers fibrinogen, CRP, APCS, THPO and VWF separate in the pathway analysis although several recent studies show their local origin and role in the lung.

## Discussion

It is generally believed that a controlled inflammatory response is beneficial but it can become detrimental if dysregulated. Irrespective of the cause, inflammation has evolved as an adaptive response for restoring homeostasis [25]. We have previously shown that inhalation of carbonaceous nanoparticles causes cellular stress responses in macrophages and epithelial cells of the lungs within few hours [26]. There are several reports showing that inhaled ultrafine particles gain rapid access to the pulmonary epithelial, interstitial and even endothelial layers of the lung and thus often escape the clearance process of the respiratory system [27-29]. In this study we aimed to contribute to a better understanding of the molecular mechanism of restoration of lung tissue homeostasis following CNP challenge while in parallel PMN influx related inflammation is being resolved. We could show that a mouse strain with robust lung physiology (C3), when exposed to a moderately toxic CNP (carbon black, Printex90) exhibits a dose dependent PMN influx1 day after instillation (358.24 ± 41.91 × 10E3 PMNs/50 μg). C3 mice were also able to completely resolve the neutrophilic influx (105.53 ± 12.37 × 10E3 PMNs/20 μg/day 1 versus 9.23 ± 3.05 × 10E3 PMNs/sham control ) by the 7^th ^day post instillation of 20 μg CNPs. Besides classical lung inflammation analysis, we applied a comprehensive panel of protein markers, primarily representing soluble proteins involved in cell-cell communication. For our time course study we have chosen a particle dose of 20 μg, related to a particle surface area of 54 cm^2^/mouse which has been previously shown to cause a significant level of pulmonary inflammation, characterized by 25% BAL PMNs in BALB/cJ mice, and which is well below any saturation conditions [12]. Similarly for the C3H/HeJ (C3) strain used here, the dose-response relationship (response expressed as BAL PMN cell number) from 5 over 20 to 50 μg CNP revealed a linear shape (R^2 ^= 0, 99).

The "time course" markers (Categories 2A and 2B) have upon immediate induction after at day 1, been detected at elevated levels also on day 7 following CNP instillation. In this context, it is noticeable that protein concentrations of markers representing processes of tissue homeostasis like fibrinogen, CRP, APCS, FGF2, vWF, THPO, VEGF, and EDN1 (sorted by Category 2B, A criteria, as shown in Figure 2a and 3 ) are in particular abundant in the inflammation resolution phase, from day 3 till day 7, where as peak-concentrations for pro-inflammatory proteins (like CXCl1, -2, -10, CCL2, -12, -22, IL6, -11 and IFNγ) known to regulate acute inflammation with leukocyte activation and recruitment are expectedly detected no later day 1 after CNP instillation. Extensive literature based analysis of these time course markers revealed astrong association of IL1B and IL18, suggesting them as the key players; and VEGF as the main down stream factor controlled through a EDN1, FGF2 network. This whole set of events seems to be modulated by a number of other factors also detected at elevated levels at day 7, namely RANTES (CCL5); MCSF (CSF1); MIP1α (CCL3); MIP1β (CCL4); MIP1γ (CCL9), FGF2, Eotaxin (CCL11); GCP2 (CXCL5); IL-18; Lymphotactin (XCL1) through a complex regulatory network. In this context it is important to mention that proteomics based studies are functionally closer than transcript based studies. Although our study is proteomics based but is limited to a finite number of proteins representative of some pathways. The striking coincidence at day 7 of two events occurring simultaneously: (i) complete resolution of inflammation at cellular level along with any evident tissue damage, and (ii) detection of elevated or even increased levels of several proteins usually classified as proinflammatory markers, suggests their differential function at this later time point more in the direction of their role in promoting tissue homeostasis. Therefore in the following section we have discussed the possible molecular cascade which we could associate to tissue homeostasis and defense response in C3 mice. However we are aware of the fact that only controlled intervention studies aimed at revealing mechanistic insights at specific time points will precisely answer the causation of possible pathological situations that may arise due to disruption of this cascade and the fact that our findings are associative at this point of time.

### IL1B and IL18 are the two likely key upstream molecules

Interleukin-1B is a master cytokine, known to be involved in initiating the innate immune response in vertebrates [30] and regulation of the expression of several other cytokines like CCL3, -4, -5, -9 and -11 [31-33]. In vitro, macrophages exposed to titanium particles release CSF1/M-CSF through upregulation of IL1B [34]. Treatment of human endothelial cells with IL1B enhances mRNA expression of the human CXCL5 homologues [35] and CXCL5 is also predominantly induced in mesenchymal cells by IL1B [36]. On a related note we have previously reported high correlation between the degree of particle exposure related acute lung inflammation (PMN numbers) and IL1B BAL concentrations in mice [12]. In vitro, macrophages exposed to titanium particles release CSF1/M-CSF through upregulation of IL1B [34]. Several studies have shown direct IL1B mediated induction of IL18 and vice versa [37,38]. IL18 can also induce a number of cytokines including CCL3 [39], CCL4 [40], CCL5 [41], CCL11 [42]. Overall, this suggests that IL1B and IL18 together play a crucial role for initiating the immediate inflammatory response to CNP. CSF1/M-CSF can regulate IL18 production by human monocytes which in turn may affect the activation and differentiation of T cells and T-cell mediated immune response [43]. From these studies the involvement of IL-1B and IL-18 during the early initiation phase of inflammation up to day 1 is well described, but less is known for their role in the subsequent resolution phase. During this time, increased IL-1B levels in the lung tissue may activate macrophages to clear the battleground such as phagocytosized neutrophis or cellular debris, as recently suggested by Al-Banna in a model of bacterial lung infection [44] On a related note a tissue protective role against bleomycin-induced lung injuries has also been described for IL-18 [45]. Since the clearance of inflammatory cells is among the first steps in resolving the inflammatory responses, the regulation of tissue protective genes by IL-1 related signalling might rather be considered for the phase from 3 to 7 days post instillation.

### Elevated levels of classical systemic markers

In the current study we detected elevated lung protein levels of CRP, APCS, fibrinogen, factor III (tissue factor), and VII, vWF, and THPO, even up to day 7. CRP is only minimally induced during inflammation in mice in contrast to humans[46], whereas serum amyloid P (APCS), the murine homologue to CRP, is strongly induced following inflammatory stimuli like endotoxin. However recent mouse studies failed to detect particle induced hepatic expression or serum release acute phase markers but rather suggest a local, pulmonary response upon inhalation [47,26]. Also in an earlier study we showed induced lung mRNA expression of acute phase proteins (serum amyloid A3, factor III, and Serpina3n/antitrypsin) upon inhalation of CNP [26]. Factor III upon interaction with lung epithelial cells exposed to asbestos particles, has been suggested to represent a potential mechanism by which particles might modulate lung remodelling related epithelial cell responses [48]. During localized injury and repair the deposition of fibrinogen beta peptide (FGB) in ECM is discussed as an important cellular response mechanism to restore tissue homeostasis and thereby protecting alveolar barrier function [49-51]. Similar implication might hold true for vWF and THPO, two factors which like fibrinogen are mainly known for their role during blood coagulation. Hence this may indicate an enduring tissue response and suggest the blood coagulation machinery to be actively engaged.

### Crosstalk between defence and homeostatic response leading to increased VEGF

As indicated in the schematic pathway model in figure 4, the proposed "defence pathway", basically represented by the inflammatory cell activating and attracting cytokines, CCL3, -4, -5, -9, -11, CXCL5, and XCL1) interacts with the proposed "homeostatic pathway", represented by VEGF, FGF2, and EDN1 via other factors (CSF1, CD40L) and also by the central players IL1B/IL18. Production of VEGF and FGF2 is reported to be enhanced by IL1B [52]. IL1B was shown to increase the release of EDN1 by primary endothelial cells in a dose dependent manner [53] and EDN1 in turn can regulate IL18 production [54]. IL18 dose dependently increase the production of VEGF [55]. FGF2 and VEGF represent important angiogenic growth factors within this network. FGF2 acts mainly by mediating VEGF production. Similar to VEGF, upon CNP instillation FGF2 lung concentrations increased dose dependently and remained elevated over time. Activated FGF2 is able to trigger the expression of CSF1 by stromal cells thus modulating the inflammatory response [56]. In our study, upon CNP exposure CSF1 remained elevated in the lung tissue, but declined to basline levels in the lavaged compartment (Figure 2). Significant higher levels of FGF2 have been noted in vascular cells following treatment with EDN1 [57,58] and cell type specific FGF2 transcript regulation by EDN1 and IL1B has been reported [59]. CSF1 can also induce VEGF production by monocytes [60]. Higher levels of VEGF, eventually induced by pulmonary EDN1 expression, contributes to pulmonary oedema formation [61] but moderately increased production of VEGF in the injured lung may also represent a protective pathway for lung injury recovery and contribute to the resolution of inflammation [62]. It seems noteworthy at this point, that we did not detect any evidence for epithelial or alveolar barrier damage upon 20 μg CNP instillation, as indicated by unchanged BAL protein concentrations (Figure 1c). Thus regardless of the significant inflammatory reaction alveolar-capillary barrier integrity was maintained in C3 mice.

VEGF, FGF2 and EDN1 are mainly involved in processes like angiogenesis, lung development, blood vessel development and cell proliferation. As suggested by our analysis (Figure 4) the cascade ends at VEGF via the FGF2 and EDN1 network. In the adult lung alterations in VEGF homeostatsis have been attributed to the pathogenesis of bronchopulmonary dyplasia, acute lung injury, emphysema and pulmonary arterial hypertension (PAH). Decreased VEGF levels resulted in the loss of its protective function on vascular endothelial cells and associated apoptosis in early acute respiratory distress syndrome (ARDS) [63,64]. VEGF also facilitates repair following lung injury by protecting the epithelial surface [65]. Therefore it is critical that an optimal regulation of VEGF signalling is maintained to protect the lung tissue following any inflammatory response. Based on our analysis we propose that a molecular cascade as represented in figure 4 is active at day 7 post instillation in C3 mice to counter any tissue disintegrity and to maintain tissue homeostasis.

To conclude, from this study we are able to associate an orchestrated set of cellular and molecular events triggered in the lungs of C3 mice with robust lung physiology, allowing resolution of the acute inflammatory response with complete abolition of the PMN influx following i.t. instillation of 20 μg CNP particles to restore the tissue homeostasis. Pathway and network modeling (Ingenuity Pathway Analysis) suggests that the major upstream events in this protective cascade are IL1B and IL18 which through a complex interaction of several other factors end up at eleveated VEGF levels via EDN1 and FGF2. Considering the pleiotrophic functional nature of many of the chemokines it is interesting to note that elevated levels of CCL9, CCL3, CCL4, CXCL5, CCL5 and XCL1 at day 7 did not cause an inflammatory response and thereby indicate their dual characteristics: in imparting acute inflammatory response (till day 1) and later on possibly in restoring tissue homeostasis. However in this study relatively smaller changes in protein concentrations (like 1.5/1.6 folds) although statistically significant warrant careful interpretation for their biological significance. It needs to be investigated if mouse strains with unfavorable lung physiology are able to resolve CNP induced inflammation through a similar or different mechanism or they succumb to such a challenge. Modern but elaborate approaches of mouse genetics, allowing controlled intervention, cell specific and at selected time points will certainly help to reveal how these factors modulate the entire process. First of all, comparing mouse strains with a distinctive capability or kinetic to restore tissue homeostasis upon CNP induced lung inflammation, should give important information about the functional contribution of the here discussed proteins for pro-resolving and/or tissue protective pathways.

## Methods

### Particles

For CNP instillation Printex 90 particles obtained from Degussa (Frankfurt, Germany) were used as described earlier [12]. Vials of 5 μg, 20 μg and 50 μg CNP particles were prepared just before use by suspending in pyrogene-free distilled water (Braun, Germany). The suspension of particles was sonicated on ice for 1 min prior to instillation, using a SonoPlus HD70 (Bachofer, Berlin, Germany) at a moderate energy of 20 Watt. We favour the use of distilled water for suspension of particles because the salt content of phosphate-buffered saline (PBS) causes rapid particle aggregation comparable to the "salting-out" effect [66] and thus eliminates consistent instillation conditions. Zeta potential (33 mV) and intensity weighted median dynamic light scattering diameter (0.17 μm) were measured for the printex 90 particles in a pyrogen free distilled water suspension at a concentration of 20 μg/50 μl using Zetatrac (Model NPA151— 31A; Particle Metrix GmbH, Meerbusch, Germany). The particle suspension was prepared under identical conditions used for mouse intra tracheal instillation.

### Mouse procedures

#### Animals

This study was approved by the Bavarian Animal Research Authority (Reference No: 55.2-1-54-2531-115-05). All the female C3H/HeJ (C3) animals were purchased from the Jackson Laboratories (Bar Harbour, ME USA) at the age of 8 weeks at the same time. The animals were housed and acclimatized at the animal facility of Helmholtz Zentrum München under specific pathogen free conditions according to the Eurpoean Laboratory Animal Science Association Guidelines [67] for at least 4 weeks. Food and water were available *ad libitum*. The experiments were performed in 12-14 weeks old animals. Experimental groups were age matched and the age of 12-14 weeks was considered for this study so as to exclude the effect of any lung developmental events that may interfere with susceptibility. By the age of 10 weeks lung development is completed in mouse and the lung assumes a completely developed, fully grown and matured structure.

Mice were anesthetized by intraperitoneal injection of a mixture of xylazine (4.1 mg/kg body weight) and ketamine (188.3 mg/kg body weight). The animals were then intubated by a nonsurgical technique [68]. Using a bulbheaded cannula inserted 10 mm into the trachea, a suspension containing 5, 20, or 50 μg CNP (Printex90) particles, respectively, in 50 μL pyrogene-free distilled water was instilled, followed by 100 μL air.

#### Experimental Design

Seven experimental groups were selected which included cage control, sham exposed, and CNP exposed (5 μg/day 1, 20 μg/day 1, 20 μg/day 3, 20 μg/day 7, 50 μg/day 1) by intratracheal (i.t.) instillation. Cage control animals were not instilled, and sham animals received 50 μL pure distilled water (vehicle). The animal groups were designed so as to obtain an acute dose-response relationship [5 μg/day 1, 20 μg/day 1 and 50 μg/day 1] and also to get a time course response [20 μg/ay1, 20 μg/day 3, 20 μg/day 7] following i.t. instillation. Therefore 5 groups were exposed to particles and 2 groups served as control (cage control and sham exposed). Each of the seven experimental groups consisted of 11 animals. 7 animals were lavaged and tissue samples from 4 mice were collected for protein analysis. 4 non-lavaged animals were used for histopathology. Lavaged lungs were immediately frozen in liquid nitrogen following dissection and stored at -80°C until next procedures for molecular analysis. Animals were treated humanely and with regard for alleviation of suffering.

#### Bronchoalveolar lavage (BAL) and analysis

On day 1/day 3/day 7 (as per experimental design) after instillation, mice were anesthetized by intraperitoneal injection of a mixture of xylazine and ketamine and sacrificed by exsanguination. BAL was performed accordingly (i.e. day 1/day 3/day 7 after instillation) by cannulating the trachea and infusing the lungs 10 times with 1.0 mL PBS without calcium and magnesium, as described previously [69]. The BAL fluid from lavages 1 and 2 and from lavages 3-10 were pooled and centrifuged (425 *g*, 20 min at room temperature). The cell-free supernatant from lavages 1 and 2 were pooled and used for biochemical measurements such as total protein and panel assays. The cell pellet was resuspended in 1 mL RPMI 1640 medium (BioChrome, Berlin, Germany) and supplemented with 10% fetal calf serum (Seromed, Berlin, Germany); the number of living cells was determined by the trypan blue exclusion method. We performed cell differentials on the cytocentrifuge preparations (May-Grünwald- Giemsa staining; 2. 200 cells counted). We used the number of polymorphonuclear leukocytes (PMNs) as a marker of inflammation. Total protein content was determined spectrophotometrically at 620 nm, applying the Bio-Rad Protein Assay Dye Reagent (no. 500-0006; BioRad, Munich, Germany). We analyzed 50 μl BAL/mouse to assess panel assays. We have not observed any statistically significant difference between cage control and sham exposed control animals in any of the measurements performed using BAL. Therefore we have compared all values in comparison to sham control to avoid redundancy of data.

#### Histology

4 not lavaged animals per experimental group were used for histological analysis. Mice were sacrificed by overdose of Ketamin and the lungs were inflation-fixed at a pressure of 20 cm H_2_O by instillation of phosphate buffered 4% formaldehyde. Three cross slices of the left lobe and slices of each right lobe (4 lobes) were systematically selected and embedded in paraffin, and 4 μm thick sections were stained with hematoxylin and eosin. The sectioned were then studied by light microscopy.

#### Protein panel assays

Total lung homogenate was prepared using 50 mM Tris-HCL with 2 mM EDTA, pH 7.4 as the lysis buffer (1000 μl) from 4 animals/experimental group. Using the Rodent MAP™ version 2.0 of the Rules Based Medicine (Austin, Texas) a panel of mostly proinflammatory and inflammatory markers were analyzed from total lung homogenate and BAL. The BAL and lung homogenate were always taken from the same animals to avoid any inter animal variation. BAL of the 4 animals/group was pooled for the measurement and only the markers equal to/above (≥) the sensitivity level were considered. Sensitivity level is the least detectable dose (LDD) as provided by Rules Based Medicine. We consider in pooled samples the markers below sensitivity levels to be not reliable due to lack of scope for reproducibility in multiple independent samples. However, in the lung homogenate markers below LDD were also considered for analysis and discussion as we could measure samples from 4 independent animals/group. In all cases a strong homogeneity of data were observed.

Additionally three more markers hemoxygenase-1 (HO-1; Stressgen Catalog # 960-071), osteopontin (SPP1; Stressgen Catalog # 900-090A) and liopcallin-2 (LCN2; R&D Systems Catalog # DY1857) were assayed from the same samples using the respective ELISA kit.

We have not observed any statistically significant difference between cage control and sham exposed control animals in any of the measurements performed using BAL and lung homogenate. Therefore we have compared all values in comparison to sham control to avoid redundancy of data.

#### Statistics

A two-way analysis of variance (ANOVA) was used to analyze differences between control and various exposure groups. P values less than 0.05 were considered as statistically significant. All computations were done by the software packages Statgraphics plus v5.0 (Manugistics, Rockville, MD) and SAS V9.1 (Cary, NC). Data are presented as arithmetic mean values of n observations ± the standard error (SE).

### Heatmaps and pathway analysis

Protein expression data from lung tissue (means, n = 4) and BALF (pools from 4 animals) were used for heatmap generation. Protein concentrations were normalized to the highest value for each protein (set to equal 1) and the resulting values were used as input for heatmap generation with CARMAweb [70].

Ingenuity software was used to generate interaction networks for selected regulated proteins. Only direct interactions between input genes were considered for network construction.

## Competing interests

The authors declare that they have no competing interests.

## About the authors

From the Comprehensive Pneumology Center, Institute Lung Biology and Disease (KG, SU, ST, KP HS and TS); Institute of Experimental Genetics (MI, JB), Helmholtz Zentrum Munich: German Research Center for Environmental Health; Department of Pediatrics Pneumology and Immunology, Charite Universitätsmedizin Berlin, Germany, ^5^Department of Pediatrics, Ruhr-University Bochum, Germany (EH).

## Authors' contributions

KG, SU, EH, HS and TS conceived and designed the experiments. KG, SU, ST, KP performed the experiment; KG, SU, ST, MI, JB, HS and TS analyzed the data; KG, SU, MI, HS and TS wrote the manuscript.

Drs. HS and TS contributed equally to this article

All authors read and approved the final manuscript.

## List of abbreviations

ANOVA: analysis of variance; ARDS: acute respiratory distress syndrome; BAL: broncho alveolar lavage; C3: C3H/HeJ; CNP: carbon nanoparticles; COPD: chronic obstructive pulmonary disease; ELISA: enzyme linked immunosorbent assay; GO: gene ontology; h: hour; H_2_O: Water; IL: Interleukin; i.t: intratracheal; LDD: least detectable dose; μg: microgram; PBS: phosphate buffered saline; PMN: polymorphonuclear leucocytes; Ptx90: printex 90; SE: standard error; w.r.t: with respect to; %: per cent.

## Supplementary Material

Additional file 1 **Table S1**. List of all the analyzed proteins along with their respective gene symbol, Entrez identification number, the least detectable dose (LDD )where applicable in our measurements and the associated gene ontology classifications according to Mouse Genome Informatics (MGI) database. Click here for file
