# Supplementary material for: Pathway focused protein profiling indicates differential function for IL-1B, -18 and VEGF during initiation and resolution of lung inflammation evoked by carbon nanoparticle exposure in mice
Source: Part Fibre Toxicol. 2009 Dec 2;6:31. doi: 10.1186/1743-8977-6-31 (PMC2809500; doi:10.1186/1743-8977-6-31)
Supplement: Additional file 1 — Table S1. List of all the analyzed proteins along with their respective gene symbol, Entrez identification number, the least detectable dose (LDD )where applicable in our measurements and the associated gene ontology classifications according to Mouse Genome Informatics (MGI) database. [file 1743-8977-6-31-S1.doc]

**Table S1:** List of all the analyzed proteins along with their respective gene symbol, Entrez identification number, the least detectable dose (LDD )where applicable in our measurements and the associated gene ontology classifications according to Mouse Genome Informatics (MGI) database.

| **Marker** | **Gene Symbol** | **Entrez Gene Id** | **Associated GO terms** | **Least Detectable Dose (LDD)** | **Expression in Pooled BAL samples**  **≥LDD** | **Expression in Lung homogenate**  **≥LDD** |
| --- | --- | --- | --- | --- | --- | --- |
| Apolipoprotein A1* | *Apoa1* | 11806 | Blood vessel endothelial cell migration; cholesterol biosynthetic process | 10 ug/mL | × | × |
| CD40 antigen* | *Cd40* | 21939 | B-cell activation; Immune response (IR) | 12 pg/mL | × | √ |
| CD40 Ligand | *Cd40lg* | 21947 | B-cell differentiation, IR, Isotype switching | 92pg/mL | √ | √ |
| C Reactive Protein (CRP) | *Crp* | 12944 | Acute phase response | 0,83ug/mL | × | × |
| Epidermal Growth Factor (EGF) | *Egf* | 13645 | MAPKKK cascade; branching morphogenesis | 39pg/mL | × | × |
| Endothelin-1 | *Edn1* | 13614 | Blood vessel morphogenesis | 67pg/mL | × | × |
| Eotaxin | [*Ccl11*](http://www.ncbi.nlm.nih.gov/sites/entrez?Db=gene&Cmd=ShowDetailView&TermToSearch=20292&ordinalpos=1&itool=EntrezSystem2.PEntrez.Gene.Gene_ResultsPanel.Gene_RVDocSum) | 20292 | Chemotaxis (C); IR; Inflammatory response (Inf R) | 12pg/mL | × | √ |
| Factor VII | *F7* | 14068 | Blood coagulation | 0,96ng/mL | √ | √ |
| Fibroblast Growth Factor-9 (FGF-9) | *Fgf9* | 14180 | MAPKKK cascade; Lung development | 0,99ng/mL | × | √ |
| Fibrinogen | *Fga,Fgb,Fgg* | 14161,110135,99571 | Blood coagulation | 170ug/mL | × | √ |
| Granulocyte Chemotactic Protein-2  (GCP-2 ) | *Cxcl5* | 20311 | C; IR; Inf R | 0,025ng/mL | √ | √ |
| Granulocyte Macrophage-Colony Stimulating Factor  (GM-CSF ) | *Csf2* | 12981 | IR | 8,7pg/mL | √ | √ |
| Glutathione S-Transferase alpha*  (GST-α ) | *Gsta1-4* | 14857-14860 | ROS; NOS | 0,42ng/mL | Not detectable | Not detectable |
| Haptoglobin* | *Hp* | 15439 | [Proteolysis](http://www.informatics.jax.org/searches/GO.cgi?id=GO:0006508) | 0,64ug/mL | √ | √ |
| Interferon-gamma  (IFN-γ) | *Ifng* | 15978 | IR; positive regulation of T-cell proliferation; Inflammatory cell apoptosis; Neutrophil apoptosis; Neutrophil chemotaxis | 68pg/mL | × | × |
| IgA * | *Igh-2* | 238447 | Systemic | 1,9ug/mL | × | × |
| Interleukin-10 | *Il10* | 16153 | IR; negative regulation of B-cell proliferation; negative regulation of IL-12 production | 109pg/mL | √ | √ |
| Interleukin-11 | *Il11* | 16156 | MAPKKK cascade | 87pg/mL | × | × |
| Interleukin-12p70 | *Il12b* | 16159, 1616 | Positive regulation of T-cell proliferation | 0,57ng/mL | × | × |
| Interleukin-17* | *Il17a* | 16171 | Inf R | 0,15ng/mL | × | × |
| Interleukin-18 | *Il18* | 16173 | IR; Angiogenesis | 0,67ng/mL | √ | √ |
| Interleukin-1alpha | *Il1a* | 16175 | IR; Inf R; Angiogenesis | 45pg/mL | √ | √ |
| Interleukin-1beta | *Il1b* | 16176 | Fever; Inf R; IR; VEGF production (positive regulation); JNK cascade; Leucocyte migration; Neutrophil chemotaxis | 0,45ng/mL | √ | √ |
| Interleukin-2 | *Il2* | 16183 | IR; Negative regulation of Inf R; Negative regulation of lymphocyte proliferation; positive regulation of T-cell proliferation | 67pg/mL | × | × |
| Interleukin-3* | *Il3* | 16187 | IR; JNK cascade | 21pg/mL | Not detectable | Not detectable |
| Interleukin-4 | *Il4* | 16189 | B-cell activation; IR; cholesterol metabolic process | 74pg/mL | × | × |
| Interleukin-5 | *Il5* | 16191 | IR | 0,19ng/mL | × | √ |
| Interleukin-6 | *Il6* | 16193 | Acute phase response; IR; MAPKKK cascade; Neutrophil apoptosis | 14pg/mL | × | √ |
| Interleukin-7 | *Il7* | 16196 | Anti-apoptosis; IR; positive regulation of B-cell proliferation; positive regulation of T-cell proliferation | 0,31ng/mL | × | × |
| Inducible Protein-10  (IP-10 ) | *Cxcl10* | 15945 | C; IR; Inf R | 40pg/mL | × | √ |
| Melanoma Growth Stimulatory Activity Protein  (KC/GROalpha ) | *Cxcl1* | 14825 | IR; Inf R | 0,17ng/mL | × | √ |
| Leukemia Inhibitory Factor  (LIF) | *Lif* | 16878 | IR; MAPKKK; Stem cell maintenance; Macrophage differentiation | 44pg/mL | × | √ |
| Lymphotactin | *Xcl1* | 16963 | C; IR | 85pg/mL | × | √ |
| Monocyte Chemoattractant Protein-1  (MCP-1) | *Ccl2* | 20296 | C; IR; Inf R | 17pg/mL | √ | √ |
| Monocyte Chemoattractant Protein-3  (MCP-3) | *Ccl7* | 20306 | C; IR; Inf R | 31pg/mL | √ | √ |
| Monocyte Chemoattractant Protein-5  (MCP-5) | *Ccl12* | 20293 | C; IR; Inf R | 46pg/mL | × | √ |
| Macrophage-Colony Stimulating Factor  (MCSF) | *Csf1* | 12977 | Macrophage differentiation; Monocyte differentiation | 0,018ng/mL | √ | √ |
| Macrophage-Derived Chemokine  (MDC) | *Ccl22* | 20299 | C; IR; Inf R | 22pg/mL | √ | √ |
| Macrophage Inflammatory Protein-1alpha  (MIP-1α) | *Ccl3* | 20302 | C; IR; Inf R | 0,23ng/mL | √ | √ |
| Macrophage Inflammatory Protein-1beta  (MIP-1β) | *Ccl4* | 20303 | C; IR; Inf R | 78pg/mL | √ | √ |
| Macrophage Inflammatory Protein-1gamma  (MIP-1γ) | *Ccl9* | 20308 | C; IR | 0,074ng/mL | √ | √ |
| Macrophage Inflammatory Protein-2  (MIP-2) | *Cxcl2* | 20310 | C; IR; Inf R | 7,2pg/mL | √ | √ |
| Macrophage Inflammatory Protein-3beta  (MIP-3β) | *Ccl19* | 24047 | C; IR; Inf R | 0,47ng/mL | × | √ |
| Matrix Metalloproteinase-9  (MMP-9) | *Mmp9* | 17395 | Collagen catabolic process | 10g/mL | √ | √ |
| Myeloperoxidase  (MPO ) | *Mpo* | 17523 | Oxidative stress | 0,95ng/mL | √ | √ |
| Myoglobin* | *Mb* | 17189 | Enucleate erythrocyte differentiation | 24ng/mL | × | √ |
| Oncostatin M (OSM) | *Osm* | 18413 | MAPKKK; Apoptosis | 0,13ng/mL | × | ×! |
| RANTES  regulated upon activation, normal T cell expressed and secreted | *Ccl5* | 20304 | C; IR; Inf R | 48pg/mL | × | × |
| Serum Amyloid P  (SAP) | *Apcs* | 20219 | Response to protein stimulus | 5,4ug/mL | × | √ |
| Stem Cell Factor  SCF | *Kitl* | 17311 | Negative regulation of apoptosis; MAPKKK cascade; Stem cell | 75pg/mL | × | √ |
| Serum Glutamic-Oxaloacetic Transaminase*  (SGOT ) | *Got1* | 14718 | Amino acid metabolism | 1,9ug/mL | × | √ |
| Tissue Inhibitor of Metalloproteinase Type-1  (TIMP-1) | *Timp1* | 21857 | [erythrocyte maturation](http://www.informatics.jax.org/searches/GO.cgi?id=GO:0043249); [metalloendopeptidase inhibitor activity](http://www.informatics.jax.org/searches/GO.cgi?id=GO:0008191) | 0,18ng/mL | × | √ |
| Coagulation factor III  (Tissue Factor) | *F3* | 14066 | Blood coagulation | 0,52ng/mL | √ | √ |
| Tumor Necrosis Factor-alpha (TNF-α) | *Tnf* | 21926 | Defense response; IR; Inf R; JNK cascade; leucocyte migration | 0,14ng/mL | √ | ×! |
| Thrombopoietin  (TPO ) | *Thpo* | 21832 | Cell Proliferation | 2,7ng/mL | √ | √ |
| Vascular Cell Adhesion Molecule-1  (VCAM-1) | *Vcam1* | 22329 | Leucocyte adhesion | 19ng/mL | × | √ |
| Vascular Endothelial Cell Growth Factor  (VEGF) | *Vegfa* | 22339 | Angiogenesis; anti apoptosis; blood vessel development; lung development | 38 pg/mL | √ | √ |
| von Willebrand Factor  (vWF) | *Vwf* | 22371 | Blood coagulation | 99 ng/mL | × | ×! |
| heme oxygenase (decycling) 1* | *Ho1* | 15368 | Oxidative stress | ELISA | × | √ |
| secreted phosphoprotein 1; Osteopontin* | *Spp1* | 20750 | Antiapoptosis | ELISA | × | √ |
| lipocalin 2 | *Lcn2* | 16819 | Response to virus transport | ELISA | × | √ |
| Fibroblast growth factor -basic | *Fgf2* | 14173 | MAPKKK, Angiogenesis, Lung development, apoptosis | 0,58ng/mL | √ | √ |

* indicates proteins not showing a dose response or remained undetectable in both bronchoalveolar lavage fluid (BAL) and lung homogenate and therefore not considered for analysis. Immune response (IR); Chemotaxis (C); Inflammatory response (Inf R)
